# Prevalence of Thrombocytopenia in Pregnant Women with COVID-19: A Systematic Review and Meta-Analysis

**DOI:** 10.3390/jcm13174980

**Published:** 2024-08-23

**Authors:** María Teresa Murillo-Llorente, Ignacio Ventura, Francisco Tomás-Aguirre, Marta Defez-Martin, María Inmaculada Martín-Díaz, Sandra Atienza-Ramirez, Francisco Llorca-Colomer, Adalberto Asins-Cubells, María Ester Legidos-García, Marcelino Pérez-Bermejo

**Affiliations:** 1 School of Medicine and Health Sciences, Catholic University of Valencia, C/Quevedo nº 2, 46001 Valencia, Spain; mt.murillo@ucv.es (M.T.M.-L.); paco.tomas@ucv.es (F.T.-A.); llorfran@mail.ucv.es (F.L.-C.); ester.legidos@ucv.es (M.E.L.-G.); 2 Molecular and Mitochondrial Medicine Research Group, School of Medicine and Health Sciences, Catholic University of Valencia, C/Quevedo nº 2, 46001 Valencia, Spain; ignacio.ventura@ucv.es; 3 General and Digestive System Surgery, Joan XXIII University Hospital, C/Dr. Mallafré Guasch, 4, 43005 Tarragona, Spain; marta.defez@mail.ucv.es; 4 Primary Health Care Center Massamagrell, C/Metge Miquel Servet, 48, 46130 Valencia, Spain; martin_inmdia@gva.es; 5 Hospital Universitario Dr. Peset, Av. Gaspar Aguilar, 90, 46017 Valencia, Spain; atienza_san@gva.es; 6 Centro de Salud de L’Eliana, Departamento Arnau de Vilanova-Lliria, C/Rosales, 23, L’Eliana, 46183 Valencia, Spain; asins_ada@gva.es

**Keywords:** blood platelets, COVID-19, pregnancy, SARS-CoV-2, thrombocytopenia

## Abstract

**Background/Objectives:** Although articles and reviews have been published on the effect of SARS-CoV-2 infection on pregnancy outcomes, they show mixed results with different hypotheses, and no work has focused specifically on the prevalence of thrombocytopenia. The objective of this systematic review and meta-analysis was to synthesize previous evidence and estimate the prevalence of thrombocytopenia in pregnant women with COVID-19. **Methods:** This systematic review was conducted according to the PRISMA-2020 and MOOSE guidelines. The Medline and Web of Science databases were searched in February 2024, and a meta-analysis of the overall prevalence of thrombocytopenia in pregnant women with COVID-19 was performed. The risk of bias was assessed using the Joanna Briggs Institute checklists. A leave-1-out sensitivity analysis was performed to test for disproportionate effect. Publication bias was assessed by visual inspection of funnel plots and Egger’s test. **Results:** A total of 23 studies met the inclusion criteria, of which 8 were included in the meta-analysis. There was significant (Q = 101.04) and substantial heterogeneity among the studies (I^2^ = 93.07%). There were no quality-based exclusions from the review of eligible studies. The combined effect of the studies showed a prevalence of thrombocytopenia of 22.9% (95%CI 4.8–41.0%). Subgroup analysis revealed no statistically significant difference in the pooled prevalence of thrombocytopenia ([16.5%; 30.3%]; *p* = 0.375. Egger’s test for bias was not significant, indicating that smaller studies did not report larger estimates of prevalence (*t* = 1.01, *p* = 0.353). Moreover, no potential publication bias was found. Our results are consistent with those obtained in pregnant women without COVID-19 infection and extend those of previous reviews of the effect of COVID-19 infection on pregnancy outcomes. **Conclusions:** Infection during pregnancy does not seem to be an additional risk factor for platelet count, although monitoring platelet count in pregnant women with COVID-19 may be of great importance to determine possible therapeutic strategies, especially in emergency cases.

## 1. Introduction

SARS-CoV-2 was first detected in Wuhan following the occurrence of several cases of bilateral pneumonia. The WHO declared the outbreak a pandemic disease in March 2020 [1]. The main routes of transmission are the inhalation of respiratory droplets and aerosols in closed environments or fomites [2,3]. Vertical transmission and breastfeeding have not been demonstrated [2].

COVID-19 presents with a wide range of symptoms, from asymptomatic patients to patients with severe and even fatal symptoms. These include fever, cough, fatigue, anosmia and ageusia, as well as dysphagia, headache, diarrhea, skin involvement and arthralgias. The most severe symptoms are dyspnea, aphasia, confusion and chest pain [4,5].

**Figure 1:**
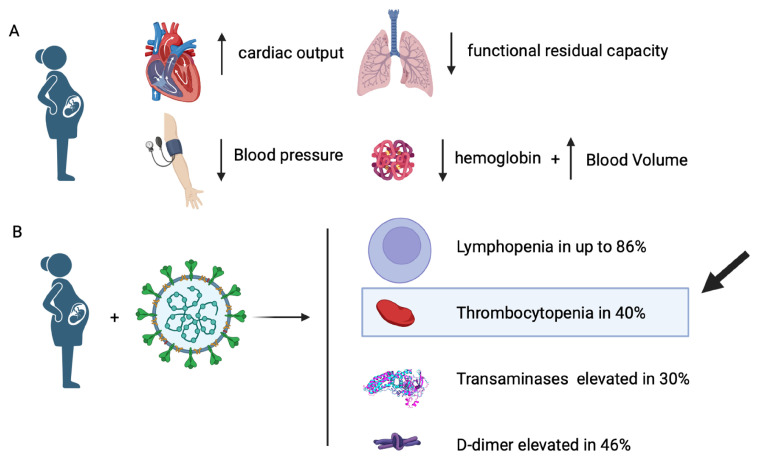
Comparison between a pregnant woman under normal conditions (**A**) and a pregnant woman with SARS-CoV-2 respiratory infection (**B**). In image A, physiological changes typical of pregnancy are highlighted, such as increased cardiac output, reduced respiratory functional residual capacity, decreased blood pressure, and variations in hemoglobin levels, accompanied by an increase in blood volume. In image B, common symptoms of SARS-CoV-2 infection during pregnancy are illustrated, including lymphopenia in 86% of cases, thrombocytopenia in 40%, and elevated transaminases in 30%, as well as an increase in D-dimer levels in 46% of cases [6,7].

The analytical values to be highlighted are the presence of lymphopenia in up to 86% of patients, thrombocytopenia in 40%, and severe thrombocytopenia in up to 5% of patients. Transaminases were elevated in 30% of patients and acute phase reactants in 60%. D-dimer was found to be elevated in 46% of SARS-CoV-2-infected patients [6,7]. Figure 1 shows a comparison between a pregnant woman under normal conditions (A) and a pregnant woman with SARS-CoV-2 respiratory infection (B).

The body of a pregnant woman undergoes a series of normal physical and psychological changes, the purpose of which is to adapt the body to the development of the fetus in the maternal uterus [8]. Blood volume increases, blood distribution varies, and cardiac output increases. Blood pressure decreases to compensate for these increases, normalizing by the end of gestation [9]. There is also a reduction in the inspiratory reserve volume, which will increase at the end of gestation due to the decrease in functional residual capacity [9]. A common symptom, occurring physiologically in up to one third of pregnant women, is dyspnea in the third trimester [10].

To compensate for vascular demands and expected blood loss during labor, total blood volume increases by one and a half liters. Erythropoietin stimulates erythrocyte production but does not fully compensate for the increase in plasma volume. This produces dilutional anemia, decreasing hemoglobin by 1–2 g/dL and increasing the mean corpuscular volume (MCV) [11]. Thrombocytopenia (platelets count less than 150 × 109/L) usually occurs in up to 12% at the time of delivery [12]. Due to the hemodilution that occurs during gestation, there is a physiological decrease in platelets, which is moderate and not related to maternal or fetal effects. However, some pathologies such as gestational thrombocytopenia, hypertensive disorders or immune thrombocytopenic purpura should be monitored and may require specific treatments [13].

Changes in pregnancy and studies of previous pandemics and influenza virus suggest that the risk of morbidity and mortality associated with SARS-CoV-2 infection may be increased in pregnant women [14,15,16]. Seventy-five percent of pregnant women are asymptomatic. The remaining symptoms are common to the general population: fever, cough, dyspnea and lymphopenia. Gastrointestinal symptoms seem to be associated with greater severity [17]. Advanced age, obesity, chronic arterial hypertension or diabetes and previous pre-eclampsia are risk factors for more severe disease [18].

Thrombocytopenia is a relatively common hematological problem during pregnancy, affecting about 7–10% of pregnant women [6,7]. The condition can have a variety of causes, including gestational thrombocytopenia, pre-eclampsia and immune thrombocytopenic purpura (ITP) [13]. During the COVID-19 pandemic, the SARS-CoV-2 virus was observed to cause thrombocytopenia in pregnant women [14,15,16]. The virus may induce platelet destruction or impair platelet production through the release of inflammatory cytokines and direct viral invasion of bone marrow cells, which could increase the risk of major bleeding, particularly during childbirth, as well as increase the likelihood of developing pre-eclampsia, a dangerous hypertensive disorder [18]. In addition, a low platelet count can complicate the management of anesthesia during labor and increase the risk of postpartum hemorrhage, posing significant risks to both mother and newborn [19,20].

Although articles and reviews have been published on the effect of SARS-CoV-2 infection on pregnancy outcomes, such as the reviews by Allotey et al. [18] and Wei et al. [21], to the best of our knowledge, no review has specifically focused on analyzing the effect of COVID-19 infection in pregnant women on the prevalence of thrombocytopenia. Therefore, the aim of this systematic review and meta-analysis was to synthesize previous evidence and estimate the prevalence of thrombocytopenia in pregnant women with COVID-19.

## 2. Materials and Methods

A systematic review was performed according to Preferred Reporting Items for Systematic Reviews and Meta-Analyses (PRISMA) 2020 [22] and Meta-Analysis of Observational Studies in Epidemiology (MOOSE) guidelines [23]. The protocol was registered under no. CRD42022332947 on PROSPERO—the International Prospective Register of Systematic Reviews (https://www.crd.york.ac.uk/prospero/; accessed on 28 February 2024). The PRISMA 2020 checklist (Supplementary Data S1) and MOOSE checklist (Supplementary Data S2) were also applied.

### 2.1. Research Question and Eligibility Criteria

The review was designed to answer the following question: “What is the prevalence of thrombocytopenia in pregnant women with COVID-19?”. We followed the PECO strategy for structuring, in which P (population), E (exposure), C (comparison), and O (outcome) were measured.

#### 2.1.1. Inclusion Criteria

- Population: pregnant women.
- Exposure: COVID-19.
- Comparator: not applicable.
- Outcome: thrombocytopenia.
- Study design: prospective or retrospective observational studies (cross-sectional, case-control, or cohort studies), case studies and case series.
- Publication language: English or Spanish.

There was no restriction on the year of publication, although, obviously, all articles were published during or after the COVID-19 pandemic period.

#### 2.1.2. Exclusion Criteria

We excluded the following works:

- Articles that did not report the prevalence of thrombocytopenia;
- Letters to the editor, personal opinions, books, book chapters, non-original reports, reviews, conference abstracts, editorials, commentaries or articles that did not contribute to or complement the study objectives;
- Articles in which the full text could not be obtained.

Case studies and case series were not excluded because of any new information that they might contribute, although they were obviously not considered in the meta-analysis.

### 2.2. Sources of Information and Search Strategy

A search for relevant articles in the Medline and Web of Science databases was performed in February 2024. The search strategy used the following Medical Subject Heading (MeSH) terms, keywords and word variants: COVID-19, SARS-CoV-2, SARS-CoV-2 variants, novel coronavirus, new coronavirus, nCoV, 2019-nCoV, severe acute respiratory syndrome coronavirus 2, severe acute respiratory syndrome, Wuhan, coronavirus, coronavirus infections, thrombocytopenia, blood platelets, thrombopenia, thrombopenia, thrombocyte, pregnancy, pregnancy complications, pregnant women, obstetrics, and gestation; these words were combined with each other using the Boolean operators “AND”, “OR” and “NOT”. Search strategies are reported in Supplementary Table S1.

In addition, a hand search of the grey literature and the bibliographic references of the included studies was carried out to include papers that might have been overlooked.

### 2.3. Selection of Studies

After eliminating duplicate articles, the initial screening of titles and abstracts was carried out, followed by a review of the full text. The initial screening was performed by two authors, M.P.-B. and M.D.-M., and the full-text review was performed by all authors.

### 2.4. Data Extraction

The following variables were extracted from the articles finally included for full-text review: the year of publication, country of study, sample and prevalence of thrombocytopenia.

### 2.5. Quality Assessment

Joanna Briggs Institute (JBI) checklists [24] appropriate for each type of study were used to assess study design and quality. These checklists have 4 response options: yes, no, unclear or not applicable. Items were scored by the number of positive responses as a percentage of the total number of possible responses, with the number of possible responses reduced by the number of ‘not applicable’ items. A score of less than 50% was considered to be of low quality. There were no exclusions from the review of eligible studies on the basis of quality. The quality assessment of each study was reviewed independently by two authors: M.P.-B. and M.D.-M. Inter-rater reliability was high. Any disagreement was discussed among the other reviewers until agreement was reached.

### 2.6. Data Synthesis and Statistical Analysis

A meta-analysis of the overall prevalence of thrombocytopenia in pregnant women with SARS-CoV-2 infection was performed. The heterogeneity of the studies was evaluated using Cochran’s Q test and the I^2^ index, accepting significant heterogeneity when *p* < 0.05 in Cochran’s Q test. The I^2^ index showed high degrees of heterogeneity when it had values greater than 50% [25]. Because of the large heterogeneity, a random-effects meta-analysis was used to calculate the overall pooled prevalence of thrombocytopenia with a 95% CI [26]. In a subgroup analysis, the effect of sample size on the prevalence of thrombocytopenia was analyzed. Two groups were created based on sample size, one with a large sample size and one with a smaller sample size, given the similar characteristics of all pregnant women with COVID-19. The results of the different studies indicating the proportion of thrombocytopenia in pregnant women with COVID-19, together with their 95% confidence intervals, were summarized.

To examine whether individual studies had a disproportionately excessive influence, a leave-1-out sensitivity analysis was applied [27]. Publication bias in the meta-analysis was detected by visual inspection of funnel plots and Egger’s test [28]. Only the 8 studies that reported the prevalence of thrombocytopenia in pregnant women with COVID-19 were included in the meta-analysis. Calculations were performed in a Microsoft Excel spreadsheet programmed by the authors.

## 3. Results

**Figure 2:**
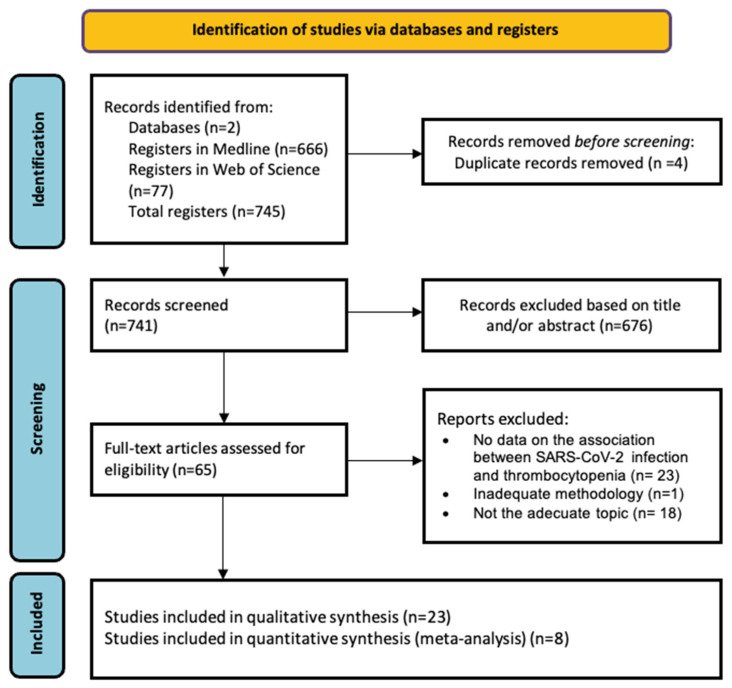
PRISMA flowchart of the study selection process.

As shown in Figure 2, the literature search identified 745 results. After eliminating duplicates and screening the remaining articles, 65 articles were selected for full-text review, of which 23 met the inclusion criteria. Table 1 summarizes the results obtained from the search of the selected studies. Supplementary Tables S2–S5 show the quality assessment of the studies.

### Results of the Meta-Analysis

**Figure 3:**
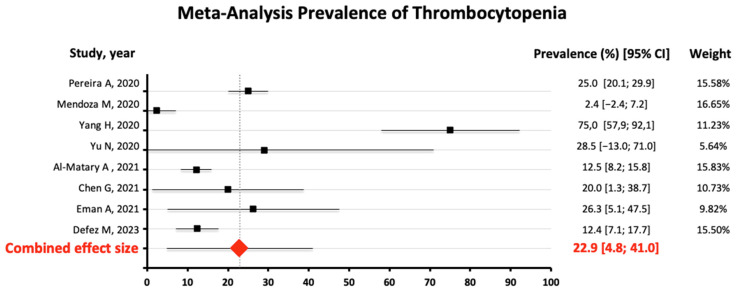
Meta-analysis of the combined prevalence of thrombocytopenia from the 8 studies [33,34,35,37,42,45,46,50].

**Figure 4:**
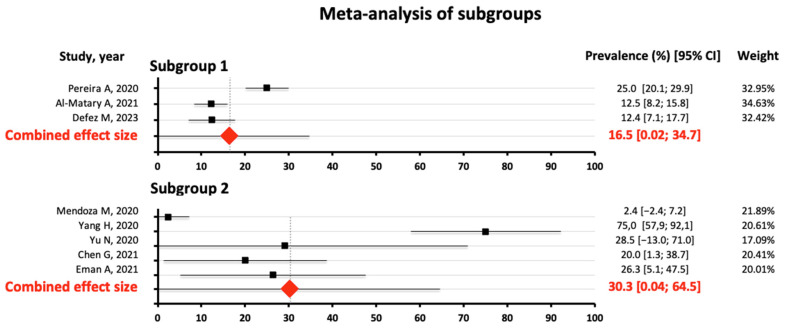
Meta-analysis of the subgroups. Subgroup 1 with 3 studies [33,42,50] and subgroup 2 with 5 [34,35,37,45,46].

**Figure 5:**
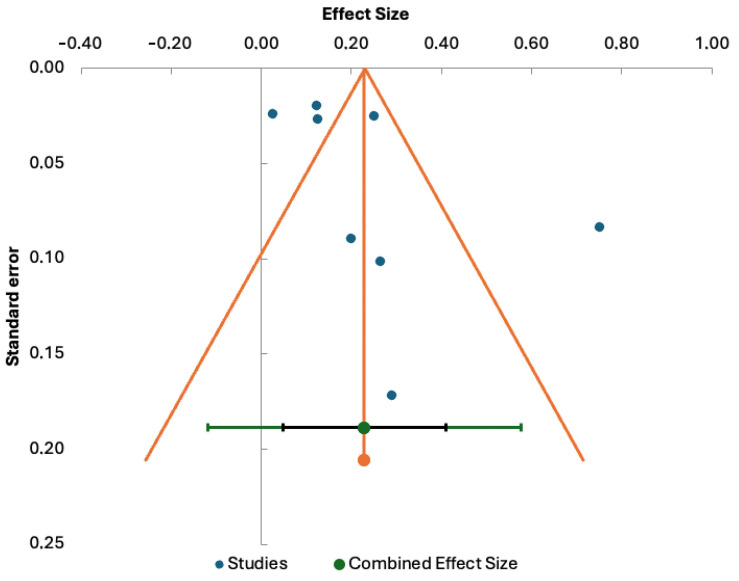
Funnel plot of the standard error by the event rate.

There were eight studies reporting the prevalence of thrombocytopenia in pregnant women with COVID-19. There was significant (Q = 101.04) and substantial heterogeneity among the studies (I^2^ = 93.07%). Because of the large heterogeneity, a random-effects model was used. Thus, the combined effect of the studies yielded a result of 22.9% prevalence of thrombocytopenia in pregnant women with COVID-19 (95%CI 4.8–41.0%) (Figure 3). Subgroup analysis revealed no statistically significant difference in the pooled prevalence of thrombocytopenia ([16.5%; 30.3%]; *p* = 0.375 (Figure 4). Egger’s test for bias was not significant, indicating that smaller studies did not report larger estimates of prevalence (*t* = 1.01, *p* = 0.353). A funnel plot (Figure 5) was used to assess potential publication bias, which did not show substantial asymmetry. Supplementary Table S6 shows the leave-1-out sensitivity analysis of the influence of a single study on the pooled prevalence of thrombocytopenia. It shows how the study by Yang et al. [35] substantially distorted the results of the meta-analysis, and its exclusion would lower the prevalence to 15.4% (95%CI 6.8–24.0%)

## 4. Discussion

The results of our meta-analysis show a pooled prevalence of thrombocytopenia of 22.9% (95% CI 4.8–41.0%). Both the subgroup analysis and the Egger test were not significant, indicating that sample size did not affect the prevalence estimates. Potential publication bias was also not found. The prevalence obtained is probably lower, closer to 15%, as shown in the sensitivity analysis after excluding Yang’s study [35], which estimated the prevalence at 75%, but it was a very limited estimate due to the really small sample size.

According to the review of studies performed, the clinical manifestations of coronavirus infection during pregnancy range from asymptomatic or mild disease to severe or fatal disease. Different studies [7,51] show that thrombocytopenia is present at a higher rate in pregnant women with severe disease compared to non-severe disease, which seems to suggest that thrombocytopenia is more prevalent in severe cases, although severe cases of thrombocytopenia have also been reported in pregnant women with non-severe COVID-19 disease [7].

Prompt recognition of thrombocytopenia, especially if severe, if present in asymptomatic or even mildly symptomatic patients with SARS-CoV-2 infection, is crucial for the provision of safe and optimal obstetric anesthetic care [32]. While reviewed studies suggest an association between SARS-CoV-2 infection and thrombocytopenia, further exploration is needed to conclusively establish the direct causal link.

Several mechanisms of thrombocytopenia production have been suggested [31,32]. On the one hand, a decrease in platelet production by direct infection of bone marrow cells, as part of secondary hemophagocytic lymphohistiocytosis, or due to the disruption of platelet release into the pulmonary circulation. Another possible cause would be increased platelet clearance by the immune system, non-specifically by the coating of immune complexes produced as part of the immune response or molecular mimicry with SARS-CoV-2. Finally, it could also be secondary to a concomitant thrombotic and consumptive process involving platelet aggregation and microthrombus formation, which is implicated in COVID-19 [31].

The proposed mechanisms of thrombocytopenia, including decreased platelet production and involvement in thrombotic processes, could benefit from a more explicit connection with the specific immune response triggered by SARS-CoV-2 infection.

In relation to the occurrence of thrombocytopenia and gestation, different studies show that when the hematologic disorder is found early in pregnancy, it undergoes a gradual decline throughout gestation [29,52]. However, Yang et al. [35] reported that the number of patients with thrombocytopenia in late pregnancy was higher than in early pregnancy.

The main risk factors reported for thrombocytopenia in pregnant women with SARS-CoV-2 infection are increased maternal age, high BMI, non-white ethnicity, pre-existing comorbidities, pre-eclampsia, gestational diabetes, and smoking [18,50]. Adverse pregnancy outcomes reported were pregnancy loss, preterm delivery, low height for gestational age, and low Apgar scores [31,53,54].

The meta-analysis collected in our work shows a combined effect of all studies of a prevalence of thrombocytopenia in pregnant women infected with SARS-CoV-2 like those described in the literature. Although some studies indicate that pregnant women with SARS-CoV-2 infection have a higher proportion of thrombocytopenia [35,49], others, on the contrary, state that SARS-CoV-2 infection is not a risk factor for a low platelet count in pregnant women [46,50,55].

Although the clinical characteristics of pregnant women with COVID-19 during pregnancy appear to be similar to those of non-pregnant adults with COVID-19 [35,37], which seems to indicate that their critical care management should be guided by the same principles as for the non-pregnant adult population, relying on effective multidisciplinary care [28], it is reasonable to assume that SARS-CoV-2 infection may be one of the causes of thrombocytopenia in pregnant women, putting them at high risk of severe problems [36]. This circumstance could support the idea that even in pregnant patients with mild COVID-19, it is essential to have a recent platelet count to guide decision making [30,50].

Likewise, the possible relationships of these patients with the occurrence of pre-eclampsia, the use of general anesthesia or the association with other hematologic disorders have been studied. Regarding pre-eclampsia, many studies report that it is a frequent cause of thrombocytopenia in pregnancy [18,30,34,39,42,56]. Others report that in cases of pregnancy with thrombocytopenia, SARS-CoV-2 infection carries an increased risk of pre-eclampsia [50]. Although no consistent published evidence has been found, it would be interesting to analyze whether this significance is bidirectional, and thrombocytopenia could follow pre-eclampsia. Due to the combination of laboratory abnormalities, such as hypertransaminasemia and thrombocytopenia, pre-eclampsia in pregnant women with COVID-19 may create a diagnostic problem, so efforts should be focused on identifying concurrent pathologies, since COVID-19 and pre-eclampsia have opposite treatment considerations [31,44]. For example, in an urgent delivery, in cased of pre-eclampsia, the use of corticosteroids should be considered to promote fetal maturation, according to the recommendations of the American College of Obstetrics and Gynecology [57], which should be weighed against the potential detrimental effects of corticosteroids in patients with COVID-19 [58].

The actual risk of general anesthesia may be considered to outweigh the theoretical risk of causing meningitis/encephalitis when performing neuraxial procedures, and, therefore, it would be advisable to perform neuraxial procedures in parturients with COVID-19 unless contraindicated [32,49]. Also, in addition to thrombocytopenia, inadequate oxygen saturation values, altered liver function, elevated C-reactive protein (CRP) and lactate dehydrogenase (LDH) levels, lymphopenia, altered neutrophil-lymphocyte ratio (NLR) and D-dimer levels were correlated with the worsening of COVID-19 [33]. In that sense, it becomes necessary to determine the specific cut-off values of aberrant hemostatic parameters associated with adverse pregnancy outcomes [59].

Finally, in terms of morbimortality throughout gestation, severe morbidity and mortality were reported mainly among pregnant women in the second and third trimesters of pregnancy. No outcomes were reported among patients with SARS-CoV-2 infection in the first trimester [53].

### Limitations

The main limitations of this study are publication bias and the heterogeneity of the studies that could overestimate disease severity due to the lack of screening of mild or asymptomatic individuals. Another possibility that may affect the results would be possible selection bias. Discrepancies in outcomes between studies finding or not finding an association between SARS-CoV-2 and thrombocytopenia in pregnant women may be attributed to methodological variations, sample sizes, and differences in the studied populations.

With respect to the meta-analysis, of all the studies analyzed in the systematic review, many were single-case or case series reports and thus could not be included in the meta-analysis, as these studies are usually inclined to report the most severe or striking cases. Although our sensitivity analysis was intended to address some of these problems, the number and sample sizes of the individual studies were too small. The articles showed much heterogeneity, so the meta-analysis results yield a pooled effect of prevalence of thrombocytopenia in pregnant women with SARS-CoV-2 infection that should be taken with caution. In summary, these findings highlight the need for further research to elucidate causal relationships while emphasizing the importance of a comprehensive approach in clinical management and suggesting potential areas for future investigation to strengthen our understanding of this complex interaction.

## 5. Conclusions

The prevalence of thrombocytopenia found in our meta-analysis aligns closely with the prevalence observed in the pregnant population without SARS-CoV-2 infection. This suggests that SARS-CoV-2 infection during pregnancy does not appear to pose an additional risk to platelet count. Identified risk factors for thrombocytopenia in pregnant women with SARS-CoV-2 infection include increased maternal age, high BMI, non-white ethnicity, pre-existing comorbidities, and specific pregnancy-related conditions such as pre-eclampsia, gestational diabetes, and smoking. These risk factors contribute to adverse outcomes such as pregnancy loss, preterm delivery, low height for gestational age, and low Apgar scores.

While our findings offer insights into the prevalence of thrombocytopenia, it is essential to acknowledge the limitations of this study. Methodological variations, sample sizes, and population differences among the analyzed studies may influence the overall interpretation. Monitoring platelet counts in pregnant women with SARS-CoV-2 infection remains crucial for determining potential therapeutic strategies, particularly in emergency cases. Further research is warranted to explore causal relationships and enhance our understanding of this complex interaction within the context of pregnancy and COVID-19.

## Figures and Tables

**Table 1:** Summary of articles admitted for review.

Reference	Country	Type of Study	Sample Total	COVID-19 Cases	Thrombocytopenia Cases (n)	Prevalence (%)
Hirshberg A, 2019 [19]	USA	Case series	5	1	1	
Tang MW, 2020 [29]	The Netherlands	Case report	1	1	1	
Gouez AL, 2020 [30]	France	Case series	3	3	3	
Kim J-H, 2020 [31]	USA	Case report	1	1	1	
Nesr G, 2020 [32]	UK	Case report	1	1	1	
Pereira A, 2020 [33]	Spain	Retrospective, observational	60	20	5	25%
Mendoza M, 2020 [34]	Spain	Prospective, observational	42	42	1	2.4%
Yang H, 2020 [35]	China	Retrospective, observational	27	4	3	75%
Braga LFB, 2020 [36]	Brazil	Case report	1	1	1	
Yu N, 2020 [37]	China	Retrospective, observational	7	7	2	29%
Schnettler WT, 2020 [38]	USA	Case report	1	1	1	
Federici L, 2020 [39]	France	Case report	1	1	1	
Zheng T, 2020 [40]	China	Case report	2	2	0	
Cao D, 2020 [41]	China	Case report	2	2	2	
Al-Matary A, 2021 [42]	Saudi Arabia	Retrospective, observational	288	288	35	12.15%
Moltner S, 2021 [43]	Canada	Case report	1	1	1	
Hansen JN, 2021 [44]	USA	Case report	1	1	1	
Chen G, 2021 [45]	China	Case Control	20	20	4	20%
Eman A, 2021 [46]	Turkey	Retrospective, observational	19	19	5	26.3%
Rawat SK, 2021 [47]	India	Case report	1	1	0	
Moses ML, 2021 [48]	USA	Case report	1	1	1	
Kumar S, 2021 [49]	India	Case series	4	4	4	
Defez M, 2023 [50]	Spain	Prospective, observational	153	153	19	12.4%

## Data Availability

Not applicable.

## Supplementary Materials

The following supporting information can be downloaded at https://www.mdpi.com/article/10.3390/jcm13174980/s1, Data S1: The PRISMA 2020 checklist; Data S2: The MOOSE checklist and Data S3: Table S1: Search strategies; Table S2: Studies evaluated using the Joanna Briggs Institute critical appraisal checklist for analytical cross-sectional studies; Table S3: Studies appraised using the Joanna Briggs Institute critical appraisal checklist for case–control studies; Table S4: Studies evaluated using the Joanna Briggs Institute critical appraisal checklist for case studies; Table S5: Studies appraised using the Joanna Briggs Institute critical appraisal checklist for case series studies; Table S6: The leave-1-out sensitivity analysis.
