# Supplementary material for: Prevalence of Thrombocytopenia in Pregnant Women with COVID-19: A Systematic Review and Meta-Analysis
Source: J Clin Med. 2024 Aug 23;13(17):4980. doi: 10.3390/jcm13174980 (PMC11396774; doi:10.3390/jcm13174980)
Supplement: Supplementary file 1 [file jcm-13-04980-s001.zip › Supplementary Data S2.pdf]

MOOSE Checklist for Meta-analyses of Observational Studies

Item No	Recommendation	Reported on Page No
Reporting of background should include		
1	Problem definition	3
2	Hypothesis statement	-
3	Description of study outcome(s)	3-4
4	Type of exposure or intervention used	4
5	Type of study designs used	4
6	Study population	4
Reporting of search strategy should include		
7	Qualifications of searchers (eg, librarians and investigators)	-
8	Search strategy, including time period included in the synthesis and key words	3
9	Effort to include all available studies, including contact with authors	3
10	Databases and registries searched	3
11	Search software used, name and version, including special features used (eg, explosion)	-
12	Use of hand searching (eg, reference lists of obtained articles)	3
13	List of citations located and those excluded, including justification	5-6
14	Method of addressing articles published in languages other than English	-
15	Method of handling abstracts and unpublished studies	-
16	Description of any contact with authors	-
Reporting of methods should include		
17	Description of relevance or appropriateness of studies assembled for assessing the hypothesis to be tested	5
18	Rationale for the selection and coding of data (eg, sound clinical principles or convenience)	-
19	Documentation of how data were classified and coded (eg, multiple raters, blinding and interrater reliability)	-
20	Assessment of confounding (eg, comparability of cases and controls in studies where appropriate)	-
21	Assessment of study quality, including blinding of quality assessors, stratification or regression on possible predictors of study results	4 & Supplementary tables S2 to S5

22	Assessment of heterogeneity	13
23	Description of statistical methods (eg, complete description of fixed or random effects models, justification of whether the chosen models account for predictors of study results, dose-response models, or cumulative meta-analysis) in sufficient detail to be replicated	4
24	Provision of appropriate tables and graphics	Figures 2 to 5 & Supplementary tables S2 to S5
25	Graphic summarizing individual study estimates and overall estimate	Figures 3 & 4
26	Table giving descriptive information for each study included	Table 1
27	Results of sensitivity testing (eg, subgroup analysis)	Figure 4 & Supplementary table S6
28	Indication of statistical uncertainty of findings	6
Reporting of discussion should include		
29	Quantitative assessment of bias (eg, publication bias)	7
30	Justification for exclusion (eg, exclusion of non-English language citations)	5
31	Assessment of quality of included studies	Supplementary tables S2 to S5
Reporting of conclusions should include		
32	Consideration of alternative explanations for observed results	9
33	Generalization of the conclusions (ie, appropriate for the data presented and within the domain of the literature review)	9
34	Guidelines for future research	10
35	Disclosure of funding source	10

Source: Stroup DF, Berlin JA, Morton SC, Olkin I, Williamson GD, Rennie D, Moher D, Becker BJ, Sipe TA, Thacker SB. Meta-analysis of observational studies in epidemiology: a proposal for reporting. Meta-analysis Of Observational Studies in Epidemiology (MOOSE) group. JAMA. 2000 Apr 19;283(15):2008-12. doi: 10.1001/jama.283.15.2008.
