# Supplementary material for: Prevalence of Thrombocytopenia in Pregnant Women with COVID-19: A Systematic Review and Meta-Analysis
Source: J Clin Med. 2024 Aug 23;13(17):4980. doi: 10.3390/jcm13174980 (PMC11396774; doi:10.3390/jcm13174980)
Supplement: Supplementary file 1 [file jcm-13-04980-s001.zip › Supplementary Data S3.pdf]

Prevalence of Thrombocytopenia in Pregnant Women with COVID-19: A Systematic Review and meta-analysis

Supplementary Material

Table S1. Search strategies

**Pubmed**	(("COVID-19"[Mesh] OR "SARS-CoV-2"[Mesh] OR "SARS-CoV-2 variants" [Supplementary Concept] OR SARS-CoV-2[tiab] OR "novel coronavirus"[tiab] OR "new coronavirus"[tiab] OR nCoV[tiab] OR COVID-19[tiab] OR covid19[tiab] OR 2019-nCoV[tiab] OR 2019nCoV[tiab] OR "severe acute respiratory syndrome coronavirus 2"[tiab] OR "severe acute respiratory syndrome"[tiab]) OR ((wuhan[tiab] OR 2019[tiab] OR 19[tiab]) AND ("coronavirus"[Mesh] OR "coronavirus infections"[Mesh] OR coronavirus*[tiab]))) AND ("Thrombocytopenia"[Mesh] OR "Blood Platelets"[Mesh] OR "Thrombocytopeni*"[tw] OR "Thrombocytopaeni*"[tw] OR "thrombopenia"[tw] OR "thrombopaenia"[tw] OR "platelet*"[tw] OR "thrombocyte"[tw]) AND ("Pregnancy"[Mesh] OR "Pregnancy Complications"[Mesh] OR "Pregnant Women"[Mesh] OR "Obstetrics"[Mesh] OR "pregnan*"[tw] OR "obstetric*"[tw] OR "gestation"[tw] OR "gestant*"[tw]) AND 2020[PDAT]:2024[PDAT]
**Web of Science**	TS=(("COVID-19" OR "SARS-CoV-2" OR "SARS-CoV-2 variants" OR "novel coronavirus" OR "new coronavirus" OR “nCoV” OR “covid19” OR “2019-nCoV” OR "severe acute respiratory syndrome coronavirus 2" OR "severe acute respiratory syndrome" OR ((“wuhan” OR “2019” OR “19”) AND ("coronavirus" OR "coronavirus infections"))) AND (("Thrombocytopenia" OR "Blood Platelets" OR "thrombopenia" OR "thrombopenia" OR "thrombocyte") AND ("Pregnancy" OR "Pregnancy Complications" OR "Pregnant Women" OR "Obstetrics" OR "pregnant" OR "obstetric" OR "gestation" OR "gestant")))

Table S2. Studies evaluated using the Joanna Briggs Institute critical appraisal checklist for analytic cross-sectional studies.

Study	Were the Criteria for Inclusion in the Sample Clearly Defined?	Were the Study Subjects and the Setting Described in Detail?	Was the Exposure Measured in a Valid and Reliable Way?	Were Objective, Standard Criteria Used for Measurement of the Condition?	Were Confounding Factors Identified?	Were Strategies to Deal with Confounding Factors Stated?	Were the Outcomes Measured in a Valid and Reliable Way?	Was Appropriate Statistical Analysis Used?	Score out of 8 (100%)
Pereira A, 2020 [33]	Y	Y	Y	Y	NA	NA	Y	Y	6/6 (100%)
Mendoza M, 2020 [34]	Y	Y	Y	Y	Y	Y	Y	Y	8/8 (100%)
Yang H, 2020 [35]	Y	Y	Y	Y	N	N	Y	Y	6/8 (75%)
Yu N, 2020 [37]	Y	Y	Y	Y	Y	Y	Y	Y	8/8 (100%%)
Al-Matary A, 2021 [42]	Y	Y	Y	Y	U	NA	Y	Y	6/7 (85.7%)
Eman A, 2021 [46]	Y	Y	Y	Y	U	N	Y	Y	6/7 (85.7%)

Y = Yes. N = No. U = Unclear.

Table S3. Studies appraised using the Joanna Briggs Institute critical appraisal checklist for case-control studies.

	Were the groups comparable other than the presence of disease in cases or the absence of disease in controls?	Were cases and controls matched appropriately?	Were the same criteria used for identification of cases and controls?	Was exposure measured in a standard, valid and reliable way?	Was exposure measured in the same way for cases and controls?	Were confounding factors identified?	Were strategies to deal with confounding factors stated?	Were outcomes assessed in a standard, valid and reliable way for cases and controls?	Was the exposure period of interest long enough to be meaningful?	Was appropriate statistical analysis used?	Score Out of 10 (100%)
Chen G, 2021 [45]	Y	Y	Y	Y	Y	Y	U	Y	Y	Y	9/10 (90%)

Y = Yes. N = No. U = Unclear.

Table S4. Studies evaluated using the Joanna Briggs Institute critical appraisal checklist for case studies.

Study	Were patient's demographic characteristics clearly described?	Was the patient's history clearly described and presented as a timeline?	Was the current clinical condition of the patient on presentation clearly described?	Were diagnostic tests or assessment methods and the results clearly described?	Was the intervention(s) or treatment procedure(s) clearly described?	Was the post-intervention clinical condition clearly described?	Were adverse events (harms) or unanticipated events identified and described?	Does the case report provide takeaway lessons?	Score out of 8 (100%)
Tang MW, 2020 [29]	Y	Y	Y	Y	Y	Y	Y	Y	8/8 (100%)
Kim J-H, 2020 [31]	Y	Y	Y	Y	Y	Y	Y	Y	8/8 (100%)
Nesr G, 2020 [32]	Y	Y	Y	Y	Y	Y	Y	Y	8/8 (100%)
Braga LFB, 2020 [36]	Y	Y	Y	Y	Y	Y	Y	Y	8/8 (100%)
Schnettler WT, 2020 [38]	Y	Y	Y	Y	Y	Y	Y	Y	8/8 (100%)
Federici L, 2020 [39]	Y	Y	Y	Y	Y	Y	Y	Y	8/8 (100%)
Zheng T, 2020 [40]	Y	Y	Y	Y	Y	Y	Y	Y	8/8 (100%)
Cao D, 2020 [41]	N	Y	Y	Y	Y	Y	Y	Y	7/8 (87,5%)
Moltner S, 2021 [43]	Y	Y	Y	Y	Y	Y	Y	Y	8/8 (100%)
Hansen JN, 2021 [44]	N	Y	Y	Y	Y	Y	Y	Y	7/8 (87,5%)
Rawat SK, 2021 [47]	N	Y	Y	Y	Y	Y	Y	Y	7/8 (87,5%)
Moses ML, 2021 [48]	N	Y	Y	Y	Y	Y	Y	Y	7/8 (87,5%)

Y = Yes. N = No. U = Unclear.

Table S5. Studies appraised using the Joanna Briggs Institute critical appraisal checklist for case series studies.

	Were there clear criteria for inclusion in the case series?	Was the condition measured in a standard, reliable way for all participants included in the case series?	Were valid methods used for identification of the condition for all participants included in the case series?	Did the case series have consecutive inclusion of participants?	Did the case series have complete inclusion of participants?	Was there clear reporting of the demographics of the participants in the study?	Was there clear reporting of clinical information of the participants?	Were the outcomes or follow up results of cases clearly reported?	Was there clear reporting of the presenting site(s)/clinic(s) demographic information?	Was statistical analysis appropriate?	Score Out of 10 (100%)
Hirshberg A, 2020 [19]	Y	Y	Y	U	U	Y	Y	Y	Y	Y	8/10 (80%)
Gouez AL, 2020 [30]	Y	Y	Y	U	U	N	Y	Y	Y	NA	6/9 (66.7%)
Kumar S, 2021 [49]	Y	Y	Y	U	U	N	Y	Y	Y	NA	6/9 (66.7%)

Y = Yes. N = No. U = Unclear.

Table S6. Sensitivity analysis of leave-1-out.

Study omitted	Prevalence of Thrombocytopenia (%) [95% CI]
Omitting Pereira A, 2020 [33]	22.6 [0.1; 44.7]
Omitting Mendoza M, 2020 [34]	26.7 [6.8; 46.5]
**Omitting Yang H, 2020 [35]**	**15.4 [6.8; 24.0]**
Omitting Yu N, 2020 [37]	22.6 [1.8; 43.4]
Omitting Al-Matary A, 2021 [42]	25.7 [4.1; 47.3]
Omitting Chen G, 2021[45]	23.3 [1.9; 44.8]
Omitting Eman A, 2021[46]	22.6 [1.3; 43.9]
Omitting Defez M, 2023 [50]	25.3 [3.8; 46.9]

[1] Bold result indicate studies that substantially detracts from the results of the meta-analysis.
